# Digital Rehabilitation Program for Breast Cancer Survivors on Adjuvant Hormonal Therapy: A Feasibility Study

**DOI:** 10.3390/cancers16234084

**Published:** 2024-12-05

**Authors:** Wing-Lok Chan, Yat-Lam Wong, Yin-Ling Tai, Michelle Liu, Bryan Yun, Yuning Zhang, Holly Li-Yu Hou, Dora Kwong, Victor Ho-Fun Lee, Wendy Wing-Tak Lam

**Affiliations:** 1 Department of Clinical Oncology, School of Clinical Medicine, LKS Faculty of Medicine, The University of Hong Kong, Hong Kong; et1510@hku.hk (Y.-L.T.); yuningz@hku.hk (Y.Z.); hollyhou@hku.hk (H.L.-Y.H.); dlwkwong@hku.hk (D.K.); vhflee@hku.hk (V.H.-F.L.); 2 Department of Clinical Oncology, Queen Mary Hospital, Hong Kong; wyl813@ha.org.hk; 3 School of Public Health, LKS Faculty of Medicine, The University of Hong Kong, Hong Kong; wwtlam@hku.hk

**Keywords:** breast cancer, hormonal therapy, digital rehabilitation, mobile app-based intervention, survivor, cognition, depression

## Abstract

**Background**: Breast cancer survivors often face physical and psychological challenges, including weight gain, metabolic syndrome, and reduced quality of life. To address these concerns, a mobile app-based rehabilitation program called “THRIVE” was developed to improve physical activity, medication adherence, and health-related quality of life (HRQoL) in this population. **Methods**: This prospective, single-arm study assessed the feasibility and effectiveness of the “THRIVE” app among breast cancer survivors undergoing hormonal therapy. Participants were recruited from Queen Mary Hospital in Hong Kong between December 2022 and June 2023. Eligible survivors had completed treatment within the last five years or had stable advanced disease on hormonal therapy. Participants monitored their exercise, medication adherence, and self-care via the app and a Fitbit activity tracker for 16 weeks. Primary outcomes included recruitment, dropout, adherence rates, and safety. Secondary outcomes, measured at baseline and week 16, included physical activity intensity, HRQoL, psychological stress, body composition, and app satisfaction. **Results**: A total of 50 participants, with a median age of 53 years, completed the study. The recruitment rate was 70.4% with no dropouts. The adherence rate, measured by completing exercises recommended in the mobile app at least three times per week, was 74%. No severe adverse events were reported. While physical activity intensity showed no significant changes from baseline to week 16 (*p* = 0.24), cognitive function (*p* = 0.021), future perspective (*p* = 0.044), arm symptoms (*p* = 0.042), depression (*p* = 0.01), and anxiety (*p* = 0.004) improved. All participants reported perfect medication compliance (100%). Satisfaction with the app was high. **Conclusions**: This mobile app-based rehabilitation program demonstrated good feasibility, with satisfactory recruitment, adherence, and safety, providing valuable insights into future definitive studies.

## Simple Summary

This study explored the effectiveness of the “THRIVE” mobile app-based rehabilitation program (Version 2.2.1) for breast cancer survivors. Fifty participants, with a median age of 53, used the mobile app and Fitbit for 16 weeks to monitor their exercise, medication, and self-care. The program demonstrated a 70.4% recruitment rate, no dropouts, and a high adherence rate of 74%, indicating strong user engagement. It was found to be safe with no serious side effects. While physical activity levels did not significantly increase, there were statistically significant improvements in cognitive function, future perspective, arm symptoms, depression, and anxiety. All participants adhered to their medication schedules with the app’s reminders. The findings suggested that the “THRIVE” program is a feasible and well-accepted tool that can support the overall recovery and well-being of breast cancer survivors. These findings highlighted the potential for further studies on app-based interventions to help this population.

## 1. Introduction

Breast cancer is the most prevalent cancer among women globally, accounting for 23% of all new cancer cases in women worldwide [1]. Fortunately, over 90% of breast cancer patients are diagnosed at an early stage without any distant metastasis [2]. The improvements in screening, early detection, and advancements in treatment have significantly reduced the risk of recurrence and improved survival rates for patients with breast cancer. It is estimated that the number of breast cancer survivors will double by 2040 [3].

Despite the advancements in breast cancer management, survivors often face both physical and psychological challenges. These include anxiety, depression, fatigue, vasomotor symptoms, cognitive dysfunction, loss of bone density, sexual dysfunction, infertility, financial burden, and unemployment [4]. Particularly concerning is the association between hormonal therapy and weight gain, as well as the development of metabolic syndrome. Up to 84% of women experience weight gain after breast cancer treatments, with an average reported gain ranging from 2.5 to 5.2 kg [5,6]. Overweight and obesity are established risk factors for the development of cardiovascular diseases and other comorbid conditions [7]. Moreover, metabolic syndrome is associated with a three-fold increase in breast cancer recurrence and approximately a two-fold increase in breast cancer-specific mortality [8,9].

To address these challenges, all breast cancer survivors are recommended to engage in regular physical activity. Previous studies and meta-analyses have demonstrated the benefits of physical activity in breast cancer survivors, including positive effects on cardiorespiratory fitness, protection against weight gain, reduction in metabolic syndrome, fatigue, anxiety, and depression, and improvement in overall quality of life [10,11,12]. Both the American Society of Clinical Oncology (ASCO) and the European Society of Medical Oncology (ESMO) have published guidelines on breast cancer survivorship, emphasizing the importance of physical activity, with recommendations of at least 150 min of moderate or 75 min of vigorous aerobic exercise per week [13,14].

In addition to weight gain and metabolic syndrome, drug compliance is another important issue for breast cancer survivors. Hormone receptor-positive (HR+) breast cancer accounts for 70–83.4% of all breast cancer types [15]. Adjuvant endocrine therapy with either aromatase inhibitors or tamoxifen is the standard adjuvant treatment for early-stage breast cancer following surgery [16]. However, the side effects of hormonal therapy, including arthralgia, hot flushes, malaise, weight gain, fatigue, insomnia, and loss of appetite, contribute to non-adherence to these medications. Previous systematic reviews have reported that the discontinuation rates of hormonal therapy among breast cancer patients range from 31% to 73%, leading to a higher risk of recurrence [17].

To better support breast cancer survivors both physically and psychologically, with the goal of improving their physical activity and drug compliance, we have developed a mobile app called “THRIVE” (Appendix A). This prospective study aimed to investigate the feasibility and efficacy of this smartphone app-based rehabilitation and educational program (mobile app + fitness tracker) on exercise capacity, drug compliance, and health-related quality of life (HRQoL) in Asian breast cancer survivors receiving hormonal therapy.

## 2. Methods

### 2.1. Study Design

This was a prospective, single-arm study conducted at Queen Mary Hospital, a university-affiliated hospital in Hong Kong. The recruitment period was from December 2022 to June 2023, with each participant being followed up for 16 weeks after enrollment. Participants were asked to download the mobile app “THRIVE” (Version 2.2.1) and were provided with a Fitbit to monitor their daily exercise for a total of 16 weeks.

### 2.2. Study Participants

Eligible participants included individuals with early-stage breast cancer who had completed surgery, adjuvant chemotherapy, or radiotherapy within the past five years and were currently receiving hormonal therapy. Additionally, individuals with advanced breast cancer on hormonal therapy with stable disease were also eligible. All participants needed to own a smartphone, consent to receive notifications from the mobile app, and wear a Fitbit daily for the 16-week study. They also had to be physically fit to participate in physical activities. Patients were excluded if they had metastatic disease requiring chemotherapy, an Eastern Cooperative Oncology Group (ECOG) performance status ≥3, a diagnosed cognitive impairment (e.g., dementia), or a physical impairment that prevented participation in physical activities.

### 2.3. Procedures

Potential participants were identified by reviewing medical records against the eligibility criteria. Eligible patients were then invited to join the study by research staff at the study site. After signing the informed consent, participants underwent baseline assessments, including the collection of demographic information (age, marital status, occupation, education level), medical history (comorbidities, concomitant medications), details of endocrine therapy, menopausal status, and current physical activity levels. Baseline physical measurements such as body weight, height, waist circumference, and body composition were also obtained. 

Participants then downloaded the THRIVE mobile app and received a Fitbit for daily use throughout the 16-week study. With assistance from research staff, they set reminder alarms for exercise and medication. Weekly app reminders encouraged them to engage in physical activities by following the exercise videos and logging their activity. Participants were also prompted to set medication diary reminders to document their intake at preferred times.

### 2.4. Study App

The “THRIVE” mobile app was developed by a multidisciplinary team consisting of oncologists, breast surgeons, oncology nurses, and qualified exercise trainers, with input from an IT company experienced in creating rehabilitation apps for Asian breast cancer survivors. The THRIVE app is compatible with both Android and iOS mobile devices. The screen captions of the THRIVE app are in Supplementary File S1.

The app has three key features:

1. Exercise Diary: This section includes three 20–30-min exercise guidance videos designed by trainers for Asian breast cancer survivors. The videos feature moderate-intensity exercises targeting lymphedema control, sleep, and fatigue management. Participants were encouraged to follow these videos weekly and record their physical activity on the app. The connected Fitbit also tracked participants’ daily exercise data, including steps taken and heart rate-based intensity (active zone minutes).
2. Education and Sharing: The patient education module provided articles on breast cancer management, medication information, symptom control, and psychological support to enhance participants’ self-management knowledge and skills. The app also included survivors’ stories to encourage peer patients.
3. Medication Diary: The app sent participants daily reminders to take their medications. Participants could customize the medication timing. For those using cyclin-dependent kinases 4/6 inhibitors (CDK4/6i) such as palbociclib or ribociclib, the app included reminders about rest days.

### 2.5. Data Collection and Analysis

Participants completed assessments before and after the 16-week rehabilitation program. These assessments included questionnaires on the intensity of physical activity, health-related quality of life (HRQoL), and satisfaction with the mobile app. In addition, their physical activity and medication adherence were monitored through the Computer Management System (CMS) of the mobile app. The medications prescribed to the patients were counted and the number of medications remaining was counted to check for medication compliance. The Fitbit data, including daily step counts and active zone minutes, were collected and downloaded in CSV format from the Fitbit application programming interface via the Fitabase platform. 

#### 2.5.1. Primary Outcome Measures

The recruitment rate was calculated by dividing the number of participants successfully recruited by the number of eligible patients approached by the research staff. The number of recruited participants per month was also recorded. The dropout rate was defined as the number of participants who withdrew from the study, providing insight into participant retention. The adherence rate of the mobile app rehabilitation program was measured by the completion of the recommended exercises in the mobile app at least three times per week during the 16-week study period. 

To ensure safety during the mobile app rehabilitation program, participants were asked about any adverse events related to the exercise program, such as falls or injuries, during monthly calls from research staff. It was noted that muscle pain and fatigue were considered non-serious events.

#### 2.5.2. Secondary Outcome Measures

The intensity of physical activity was measured using the International Physical Activity Questionnaire (IPAQ) short version [18], which is a validated tool designed to assess the time participants spent on physical activities over the past seven days through a series of four questions. The Fitbit device also tracked participants’ walking steps and active zone minutes, providing longitudinal data to evaluate changes in physical activity levels.

The HRQoL was measured using two instruments: the European Organisation for Research and Treatment of Cancer Quality of Life Questionnaire (EORTC-QLQ-C30) and the Breast-23 (EORTC-QLQ-BR23) [19,20]. The EORTC QLQ-C30 is a validated and reliable 30-item questionnaire that covers various functional and symptom domains relevant to cancer patients. The EORTC QLQ-BR23 comprises 23 questions covering issues related to breast cancer survivors, such as body image, sexual functioning, and symptom experience.

Change in psychological stress was evaluated using the validated Chinese version of the Hospital Anxiety and Depression Scale (HADS) [21,22], which is a 14-item self-rating scale that assesses levels of anxiety and depression in both hospital and community settings.

Body composition was assessed by comparing baseline measurements of weight, waist circumference, muscle mass, bone mineral mass, and body fat with those recorded at the 16-week follow-up. These measurements were conducted by research staff at both baseline and week 16 to ensure objective assessment.

Drug compliance was assessed by asking patients to bring their prescribed medications to follow-up visits at week 16, where the number of remaining pills was counted. The adherence rate was calculated using the following formula: (Number of dosage units dispensed − number of dosage units remaining)/(prescribed number of dosage units per day × number of days between two visits). Additionally, patients recorded their medication usage in the mobile app, which allowed for verification of the counted pills.

Satisfaction with the mobile app was measured using the mHealth Satisfaction Questionnaire version 2, which consists of 12 items developed based on Rasch measurement theory [23].

### 2.6. Data Analysis

Categorical variables were analyzed using the Pearson chi-square test or Fisher’s exact test. Continuous variables were analyzed using the Student’s *t*-test or Wilcoxon signed-rank test, depending on their distribution. The distribution of the outcome data was assessed using normal probability plots and the Shapiro–Wilk test. For outcomes with a normal distribution, paired *t*-tests were used to compare pre- and post-assessments. For outcomes where the Shapiro–Wilk test had a *p*-value < 0.05, indicating non-normal distribution, the Wilcoxon signed-rank test was used instead.

All statistical analyses were performed using R version 5.0, with a significance level set at *p* < 0.05.

### 2.7. Criteria to Proceed with the Definitive Study

The criteria for proceeding with the randomized controlled study included a recruitment rate of over 60%, at least 20 participants recruited per month, a dropout rate of less than 30%, an adherence rate exceeding 70%, and no serious adverse events associated with the mobile app exercise program.

### 2.8. Sample Size

A total of 50 patients were recruited for this feasibility single-arm study. There is no specific equation for accurately calculating sample size in this context. However, the target sample size of 50 is based on recommendations from the literature and previous guidelines [24].

## 3. Results

### 3.1. Characteristics of the Participants

A total of 50 breast cancer survivors on adjuvant hormonal therapy were recruited from December 2022 to June 2023. All 50 participants (median age 53 years, range 34–66 years) completed the study and were included in the analysis. All the patients had early breast cancer and none of them had advanced diseases. Demographic and clinical characteristics of the participants at baseline are detailed in Table 1.

### 3.2. Primary Outcome Measures

A total of 71 breast cancer patients were approached by our research staff, resulting in a recruitment rate of 70.4%. All participants were enrolled within 7 weeks, averaging around 28 recruits per month. Fourteen patients declined to participate for various reasons, including lack of time, reluctance to wear a Fitbit, lack of storage capacity on the smartphone, and having an outdated phone that could not download the THRIVE app.

All enrolled participants completed the 16-week mobile app rehabilitation program and completed the baseline and end-of-study questionnaires, leading to a dropout rate of 0%. The adherence rate was 74%, as 37 participants completed the recommended exercises in the mobile app at least three times per week. Additionally, 45 participants (90%) reported consistent weekly use of the app, and all participants indicated that they used the Fitbit device daily. However, some participants cited reasons for non-adherence to the rehabilitation program. These reasons included lack of time, engagement in other physical activities outside the program, feeling that the activities within the app were too easy, and a lack of interest in the exercise videos.

None of the participants reported any serious adverse events during the 16-week mobile rehabilitation program. Events unrelated to the program included flu, urinary tract infection, constipation, and dyspepsia.

### 3.3. Secondary Outcome Measures

The secondary outcome measures were summarized in Table 2.

#### 3.3.1. Intensity of Physical Activity

The intensity of physical activity remained comparable between baseline (week 0) and week 16, with no statistically significant changes in MET-vigorous (480 vs. 900 MET-minutes/week, *p* = 0.334), MET-moderate (240 vs. 360 MET-minutes/week, *p* = 0.799), MET-walk (693 vs. 907.5 MET-minutes/week, *p* = 0.051), and MET-total (2204 vs. 2622 MET-minutes/week, *p* = 0.215).

From the Fitbit data, the average number of steps increased from 10,145 (±3404) at baseline to 11,288 (±2908) at week 16, but this was not statistically significant (*p* = 0.081). The average active zone minutes were also not significantly different between baseline and week 16 (51.3 min vs. 56.8 min, *p* = 0.067).

**Table 2:** Secondary outcome measures in Week 0 and 16.

	Week 0			Week 16			*p*-Value
**Intensity of Physical Activity** ‡	Mean/Median	SD/IQR	Range	Mean/Median	SD/IQR	Range	
MET-vigorous (MET-minutes/week)	480	(180, 1050)	0–1920	900	(440, 1530)	0–7680	0.334
MET-moderate (MET-minutes/week)	240	(180, 560)	60–2520	360	(200, 840)	80–3000	0.799
MET-walk (MET-minutes/week)	693	(400, 1386)	165–11,088	907.5	(462, 1460.3)	99–8316	0.051
MET-total (MET-minutes/week)	2204	(1030, 3061.5)	632.5–5199	2622	(2115.5, 3802.5)	1292–9276	0.215
**Fitbit data** §							
Steps	10,145	3573.7	1312–23,215	11,288	4485.3	14,567–24,798	0.081
Active zone minutes	41.3	35.5	12.3–95.6	56.8	48.7	10.4–156.5	0.067
**Health-Related Quality of Life**							
**EORTC-QLQ-C30**							
Global Health §	68.5	19.5	16.7–100.0	68.2	17.4	25.0–100.0	0.891
Functional Scale: Physical functioning §	81.9	12.5	46.7–100.0	84.8	12.4	48.0–99.0	0.058
Functional Scale: Role functioning §	86.7	16.1	50.0–100.0	87.0	16.9	34.0–100.0	0.883
Functional Scale: Emotional functioning §	72.8	16.8	16.7–100.0	70.2	19.6	8.3–100.0	0.229
Functional Scale: Cognitive functioning §	72.0	20.3	0.0–100.0	78.0	17.3	33.3–100.0	**0.021**
Functional Scale: Social functioning §	80.3	23.7	16.7–100.0	85.3	19.8	0.0–100.0	0.087
Symptom Scale: Fatigue §	29.6	18.2	0.0–88.9	30.7	18.2	0.0–88.9	0.574
Symptom Scale: Nausea and vomiting ‡	0.0	(0.0, 0.0)	0.0–100.0	0.0	(0.0, 0.0)	0.0–100.0	0.518
Symptom Scale: Pain ‡	25.0	(0.0, 33.3)	0.0–83.3	16.7	(0.0, 33.3)	0.0–83.3	0.718
Symptom Scale: Dyspnoea ‡	0.0	(0.0, 0.0)	0.0–33.3	0.0	(0.0, 0.0)	0.0–50.0	0.444
Symptom Scale: Insomnia ‡	33.3	(33.3, 58.3)	0.0–100.0	33.3	(33.3, 58.3)	0.0–100.0	0.376
Symptom Scale: Appetite loss ‡	0.0	(0.0, 33.3)	0.0–33.3	0.0	(0.0, 25.0)	0.0–66.7	0.785
Symptom Scale: Constipation ‡	0.0	(0.0, 33.3)	0.0–100.0	0.0	(0.0, 33.3)	0.0–100.0	0.537
Symptom Scale: Diarrhea ‡	0.0	(0.0, 33.3)	0.0–66.7	0.0	(0.0, 25.0)	0.0–66.7	0.766
Symptom Scale: Financial difficulties §	80.5	11.7	41.6–97.8	81.4	11.6	40.0–100.0	0.440
Overall score §	68.5	15.4	50.6–98.5	68.2	15.8	47.0–81.1	0.891
**EORTC-QLQ-BR23**							
Functional Scale: Body image §	75.2	20.5	33.3–100.0	77.0	22.7	16.7–100.0	0.414
Functional Scale: Sexual functioning §	84.7	15.4	50.0–100.0	85.0	15.5	66.7–100.0	0.844
Functional Scale: Sexual enjoyment §	73.3	17.8	33.3–100.0	72.0	18.3	33.3–100.0	0.322
Functional Scale: Future perspective §	53.3	22.3	0.0–66.7	58.7	23.9	0.0–100.0	**0.044**
Symptom Scale: Systemic therapy side effects §	23.9	12.5	0.0–52.4	23.5	14.5	0.0–66.7	0.828
Symptom Scale: Breast symptoms ‡	16.7	(8.3, 25.0)	0.0–66.7	20.8	(8.3, 33.3)	0.0–66.7	0.269
Symptom Scale: Arm symptoms §	27.1	20.1	0.0–77.8	22.7	18.0	0.0–88.9	**0.042**
Symptom Scale: Upset by hair loss ‡	17.7	(0.0, 33.3)	0.0–100.0	33.3	(0.0, 33.3)	0.0–100.0	0.709
**Psychological stress (HADS)** §							
Total Depression Score	9.9	3.2	2.0–17.0	8.0	3.8	2.0–18.0	**0.010**
% with abnormal depression score	80%	0.4	0.0–1.0	52%	0.5	0.0–1.0	**0.002**
Total Anxiety Score	6.8	2.6	1.0–14.0	5.4	3.2	0.0–14.0	**0.004**
% with abnormal anxiety score	42%	0.5	0.0–1.0	18%	0.4	0.0–1.0	**0.006**
**Body composition** §							
Body Weight (kg)	58.0	9.9	41.6–86.8	58.1	10.2	41.8–86.8	0.892
Height (cm)	159.8	5.1	148.5–172.5	160.0	5.1	148.5–172.5	0.164
Waist Circumference (cm)	82.6	10.8	66.0–110.0	84.0	9.9	71.0–109.5	0.314
Muscle Mass (%)	62.4	7.3	33.0–75.2	63.1	7.7	32.9–76.6	**<0.001**
Body Fat (%)	32.9	6.4	20.4–48.3	33.0	7.4	18.9–57.9	0.913
Bone Mineral Mass (kg)	3.8	0.3	3.1–4.5	3.9	0.3	3.1–4.5	**0.001**

**Notes:** ‡ Data presented as median (IQR) due to non-normal distribution. § Data presented as mean (SD) for normally distributed variables. Bold *p*-values indicate statistical significance (*p* < 0.05). IQR: Interquartile Range; SD: Standard Deviation.

#### 3.3.2. Health-Related Quality of Life (HRQoL)

The overall score of the EORTC-QLQ-C30 did not significantly change from baseline to week 16 (68.5 vs. 68.2, *p* = 0.891). However, significant differences were observed in some subscales. In the EORTC-QLQ-C30 questionnaire, the cognitive functional scale showed a statistically significant improvement, increasing from 72.0 at baseline to 78.0 at week 16 (*p* = 0.021). For the EORTC-QLQ-BR23, the future perspective functional scale increased from 53.3 to 58.7 over the 16-week period (*p* = 0.044). Additionally, the arm symptom score reduced from 27.1 at baseline to 22.7 at week 16 (*p* = 0.042), suggesting that the program effectively alleviated arm-related symptoms. Other functional and symptom scales did not show significant changes.

#### 3.3.3. Psychological Stress

**Figure 1:**
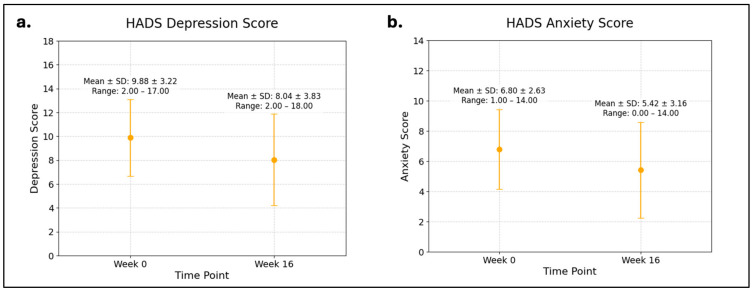
HADS (**a**) depression and (**b**) anxiety scores at Week 0 and Week 16.

Participants had significant reductions in depression scores (9.9 to 8.0, *p* = 0.01) and anxiety scores (6.8 to 5.4, *p* = 0.04) from baseline to week 16 on the HADS measure (Figure 1a,b). The percentage of participants with abnormal depression scores decreased from 80% to 52% (*p* = 0.002), and the percentage with abnormal anxiety scores decreased from 42% to 18% (*p* = 0.006) over the 16-week period.

#### 3.3.4. Body Composition

The average bone mineral mass increased from 3.8 kg at baseline to 3.9 kg at week 16 (*p* = 0.001). The muscle mass increased from 62.4% at baseline to 63.1% at week 16 (*p* < 0.001). There were no significant changes in body weight, waist circumference, muscle mass, or body fat.

#### 3.3.5. Drug Compliance

All participants utilized the drug reminder function in the app and reported a 100% compliance rate with no missed medication doses.

#### 3.3.6. Satisfaction with the Mobile App

**Figure 2:**
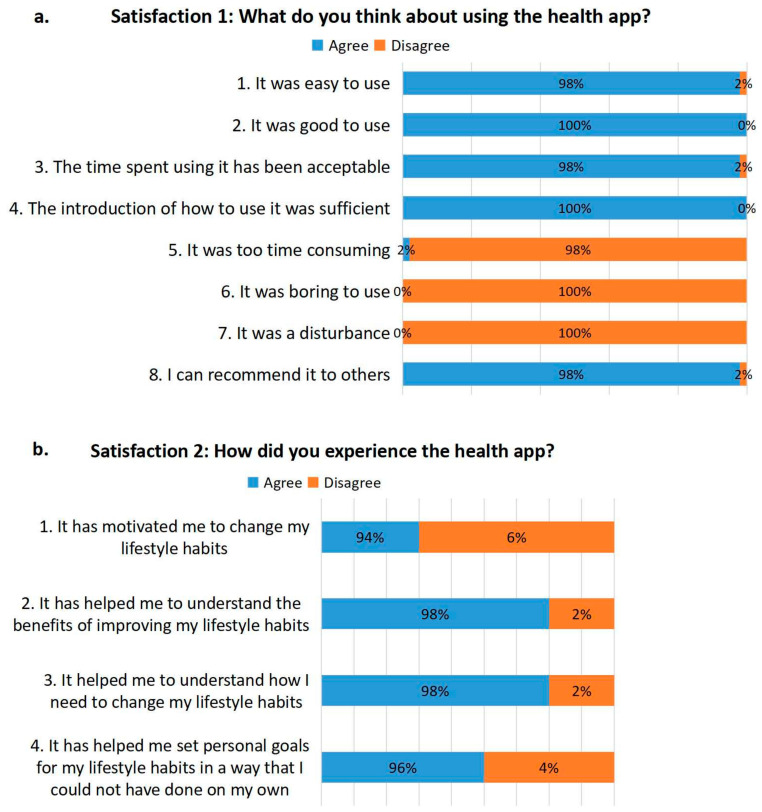
(**a**,**b**) Satisfaction on the mobile app.

The majority of the participants expressed a high level of user acceptance and satisfaction with the app (Figure 2a,b). Forty-nine participants (98.0%) found the app easy to use and the time spent on the app was acceptable. Forty-seven (94.0%) said it positively influenced their health habits and lifestyle and Forty-nine (98%) would recommend it to others. None of the participants reported that the app was boring or caused a disturbance.

## 4. Discussion

This study examined the feasibility and impact of a mobile app-based rehabilitation program (THRIVE) for breast cancer survivors. The recruitment rate was satisfactory, with a low dropout rate. The high adherence rate demonstrated that participants were highly engaged with the intervention, consistently utilizing the mobile app and fitness tracker throughout the 16-week program. The program demonstrated statistically significant improvements in cognitive function (from 72.0 to 78.0, *p* = 0.021), future perspective (from 53.3 to 58.7, *p* = 0.044), and psychological well-being (with depression scores decreasing from 9.9 to 8.0, *p* = 0.01, and anxiety scores from 6.8 to 5.4, *p* = 0.004).

The mobile app-based rehabilitation program did not significantly improve the physical activity levels of breast cancer survivors. This may be attributed to participants already having relatively high baseline levels of physical activity, averaging over 10,000 steps per day, leaving less room for improvement. The 16-week intervention duration may have been too short to observe substantial changes in physical activity behavior, as developing and sustaining long-term habits is a gradual process. Other studies on home-based exercises have durations ranging from 6 to 12 months [25]. However, a longer exercise program may result in a higher dropout rate and lower adherence.

The app’s design also lacked personalized goal setting, real-time feedback, social support, and gamification elements, which might enhance its ability to motivate and sustain physical activity. Future research should explore longer intervention periods and incorporate personalized, interactive features in mobile app-based rehabilitation programs to achieve greater improvements in physical activity levels.

Despite no improvement in physical activity levels, the rehabilitation program led to some improvements in several outcomes. Significant statistical improvements were observed in cognitive function, as measured by the EORTC-QLQ-C30 questionnaire, and in the future perspective functional scale of the EORTC-QLQ-BR23 questionnaire. These findings suggested that the intervention may have helped improve cognitive abilities and optimism about the future, which can be valuable for breast cancer survivors during the recovery process.

Cognitive complaints are a common adverse event experienced by breast cancer survivors, with an incidence as high as 50% [26,27,28]. Survivors may face difficulties with short-term memory, word-finding, executive functioning, and concentration, affecting their quality of life and work resumption. While similar mobile app-based interventions have reported mixed results in improving cognitive function and psychological well-being for cancer survivors, a systematic review by Vergani et al. found that none of the existing apps included two or more domains of cognitive functioning and were not specifically developed for breast cancer patients [29]. The positive impact on cognitive functioning observed in our study suggested that well-designed mobile app-based rehabilitation programs have the potential to address the cognitive challenges faced by breast cancer survivors. Future research should focus on the effects of mobile app interventions on different domains of cognitive functioning.

Additionally, the study found that this mobile rehabilitation program significantly reduced participants’ depression and anxiety scores. This improvement in psychological well-being could be attributed to engagement in physical activities. Previous studies have demonstrated the positive effects of physical activity on cancer and treatment-related side effects as well as their psychological health, including improvements in sleep disturbances, perceived stress, and mental health.

The participants in our study maintained 100% compliance with their prescribed endocrine therapies throughout the study period. Previous studies have shown that adherence to endocrine treatment is typically low among breast cancer survivors, with only around 60% compliance. Non-compliance with endocrine treatment can significantly increase the risk of breast cancer recurrence and impair survival. The drug reminder function within the mobile app appears to be a very effective tool in supporting medication adherence.

The study had several strengths, including the comprehensive assessment of various health outcomes and high participant engagement. The educational materials and exercise videos in the app were specifically tailored for Asian breast cancer survivors. However, the study also had limitations, such as a small sample size, a relatively short intervention duration, and a lack of a control group. Additionally, the study was conducted at a single center and only included survivors with early-stage breast cancer, with no patients having advanced disease. Future studies should consider including patients with stable disease and good performance status to enhance generalizability. While some outcomes were statistically significant, further prospective studies are needed to establish their clinical significance. Despite these limitations, the study provides valuable insights, demonstrating the potential of mobile technologies to support the multifaceted needs of breast cancer survivors. Larger-scale definitive studies are needed to further explore the effectiveness of such app-based rehabilitation programs, address the identified areas for improvement, and explore how the mobile app rehabilitation program can be generalized to breast cancer patients in different stages.

## 5. Conclusions

This feasibility study demonstrated that the mobile rehabilitation program (THRIVE) for breast cancer survivors achieved satisfactory recruitment, dropout, and retention rates, indicating that it is both feasible and well-accepted by the target population. The program was safe, with no serious adverse events reported. Although there was no significant improvement in physical activity levels, it showed promising results in enhancing cognitive function, future perspective, arm-related symptoms, psychological stress, and bone mineral mass among participants. These findings provide valuable insights for future definitive studies on mobile app-based interventions to support the holistic recovery and well-being of breast cancer survivors, especially in cognitive function rehabilitation and drug compliance.

## Figures and Tables

**Table 1:** Demographic and clinical characteristics of the participants.

Characteristics	Number of Participants (%)
Total number of participants	50 (100%)
Median age (range)	53 (34–66)
Years of survivorship (years)	0.3–5.0
Employment status	
Employed	37 (74%)
Unemployed/retired/housewife	13 (26%)
Education level	
Primary or below	0 (0%)
Secondary	21 (42%)
Diploma or above	29 (58%)
Marital status	
Single	11 (22%)
Married	36 (72%)
Divorced/widowed	3 (6%)
Menopausal status	
Pre-menopause	20 (40%)
Menopause	30 (60%)
Current use of hormonal treatment	
Tamoxifen	22 (44%)
Aromatase inhibitor	24 (48%)
LHRHa and aromatase inhibitor	4 (8%)
Stage of breast cancer at diagnosis	
Stage I	19 (38%)
Stage II	17 (34%)
Stage III	14 (28%)
Previous treatment	
Previous mastectomy	25 (50%)
Previous breast-conserving surgery	25 (50%)
Use of neoadjuvant or adjuvant chemotherapy	29 (58%)
Use of trastuzumab	14 (28%)
Received adjuvant radiotherapy	45 (90%)

Abbreviations: LHRHa: Luteinizing Hormone—Releasing Hormone analog (LHRHa).

## Data Availability

The data presented in this study are available on request from the corresponding authors. The data are not publicly available due to patients’ privacy.

## Supplementary Materials

The following supporting information can be downloaded at: https://www.mdpi.com/article/10.3390/cancers16234084/s1, File S1: Screening captions of the THRIVE app v 2.2.1.

## Appendix A

The figures show the user interfaces of the “THRIVE” app. The app aims to enhance breast cancer survivors’ rehabilitation and improve their well-being. It includes educational materials, medication reminders, exercise reminders, and recordings. The app can be downloaded on Google Play at https://play.google.com/store/apps/details?id=hku.breast.cancer&hl=en (accessed on 27 November 2024) or App Store at https://apps.apple.com/in/app/breast-cancer-survivorship/id1643853050 (accessed on 27 November 2024).
