# Supplementary figures and images for: Digital Rehabilitation Program for Breast Cancer Survivors on Adjuvant Hormonal Therapy: A Feasibility Study
Source: Cancers (Basel). 2024 Dec 5;16(23):4084. doi: 10.3390/cancers16234084 (PMC11639799; doi:10.3390/cancers16234084)

## Slide 1
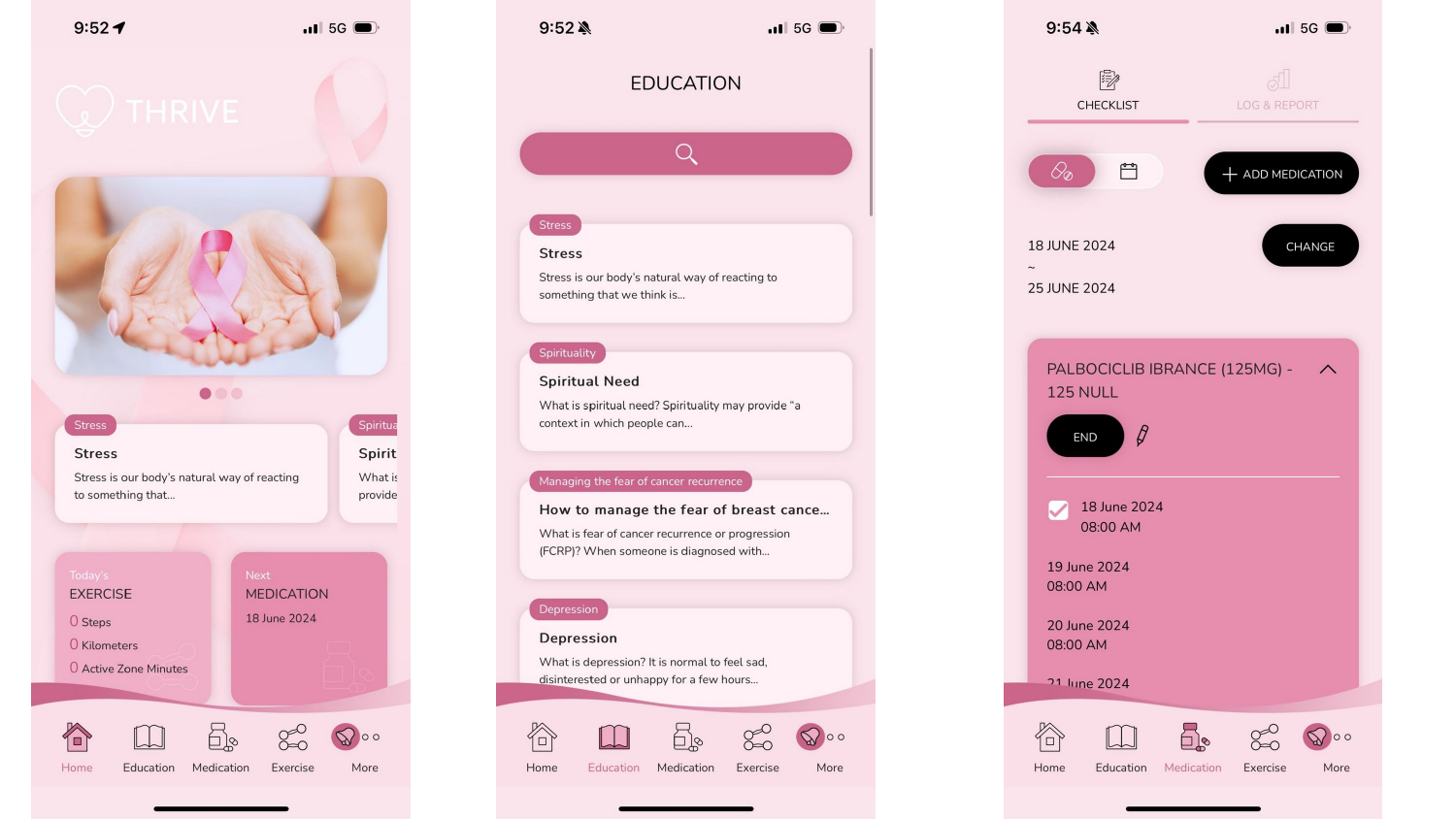

#

## Slide 2
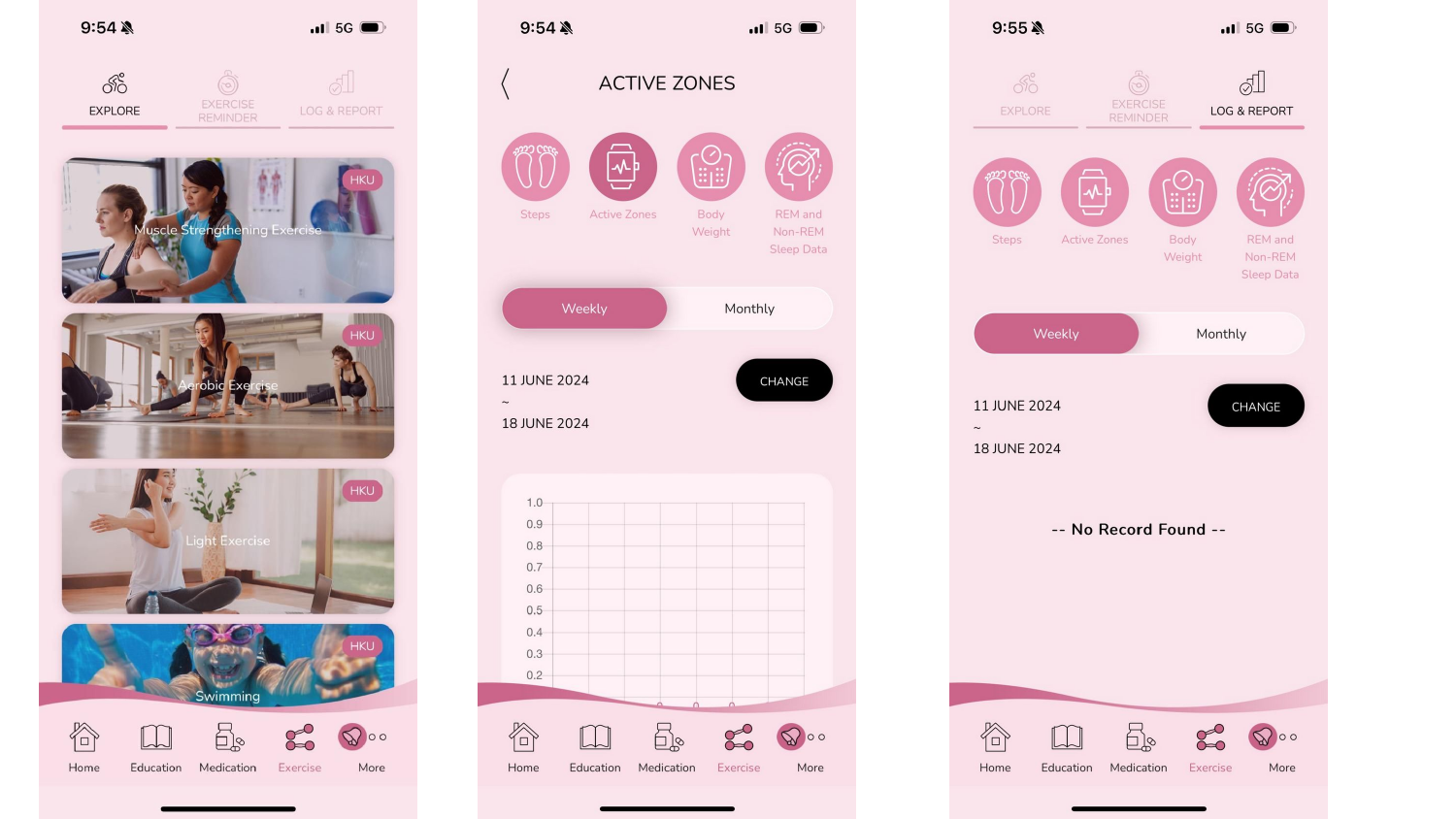

#

Supplement: Supplementary file 1 [file cancers-16-04084-s001.zip › cancers-3258672-supplementary.pptx]
